# P-1445. Evaluation of Achievement of Metabolic Goals in People with Human Immunodeficiency Virus (HIV) in the Illinois Department of Corrections (IDOC)

**DOI:** 10.1093/ofid/ofae631.1617

**Published:** 2025-01-29

**Authors:** Nivan Khair, Amber Vu, Jonathan M Ruiz, Emily N Drwiega, Mahesh C Patel, Scott Borgetti, Melissa E Badowski

**Affiliations:** University of Illinois at Chicago, Chicago, Illinois; UIC College of Pharmacy, Chicago, Illinois; University of Illinois - Chicago, Chicago, Illinois; University of Illinois Chicago, Chicago, Illinois; University of Illinois Chicago, Chicago, Illinois; University of Illinois at Chicago, Chicago, Illinois; University of Illinois Chicago, Chicago, Illinois

## Abstract

**Background:**

The UIC-IDOC clinic is an interdisciplinary telemedicine clinic addressing HIV care needs of individuals in custody. The UIC-IDOC care team makes recommendations to medical providers regarding disease states outside of HIV. HIV contributes to increases in cardiovascular (CV) risk, along with diabetes (DM), hypertension (HTN), and hyperlipidemia (HLD). Managing comorbidities in patients with HIV (PWH) can lead to an overall reduction in CV risk. The goal of this study was to provide evidence that follow-up with IDOC telemedicine clinic had positive outcomes on achieving metabolic goals in individuals in custody.

**Figure:**
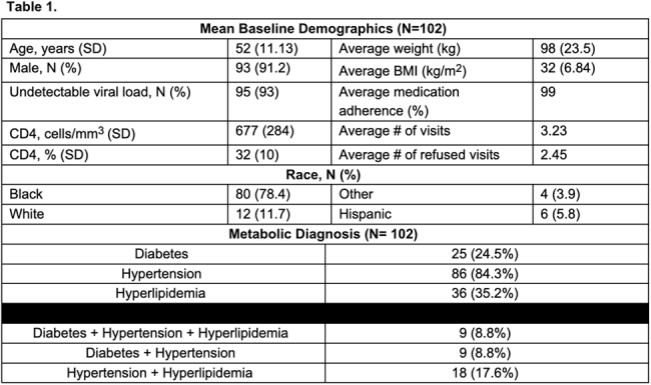

**Methods:**

This was a pilot retrospective cohort study evaluating the effectiveness of an interdisciplinary approach in achieving A1C, blood pressure, or lipid panels among PWH in custody. Adults diagnosed with HIV, on antiretroviral therapy (ART), and with a diagnosis of DM, HTN, and HLD were included between 1/1/2021 and 6/30/2022. The primary outcome evaluated the achievement of metabolic goals within 1 year compared to baseline, while the secondary outcome assessed medication optimization to guideline-recommended therapy for any diagnoses above.

**Figure:**
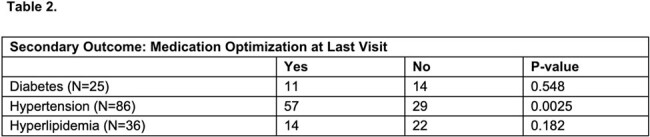

**Results:**

102 patients were included. Baseline characteristics are presented in Table 1. Notably, most PWH reported ART adherence and achieved viral suppression. For the primary outcome, there was no difference in the achievement of metabolic goals 1-year post-follow-up for all disease states, DM (p=0.347), HTN (p=0.833), and HLD (p=0.935). For our secondary outcome, there was a significant difference in the optimization of hypertensive medications by the last visit, Table 2.

**Conclusion:**

Findings from this study offer insights into optimizing HIV care delivery within correctional settings and enhancing outcomes for this patient population. Based on this data, recommendations being made by the UIC-IDOC telemedicine clinic may not be consistently implemented. However, our study shows an interdisciplinary clinic's role in the medication management of individuals in custody and the potential to assist in reducing cardiovascular risk in PWH, especially in people with HTN. Furthermore, a high degree of viral suppression was demonstrated for this study which may predict adherence to medications for metabolic diagnoses.

**Disclosures:**

**Scott Borgetti, MD**, GSK: Grant/Research Support

